# Self-Regulation Processes in Patients with Alcohol Dependence (Pilot Study)

**DOI:** 10.1192/j.eurpsy.2022.2102

**Published:** 2022-09-01

**Authors:** E. Fadeeva, A. Lanovaya

**Affiliations:** 1 National Medical Research Centre for Psychiatry and Narcology n.a. V.Serbsky Russian Federation Ministry of Health, Department Of Preventive Care In Narcology, Moscow, Russian Federation; 2 National Medical Research Centre for Psychiatry and Narcology n.a. V.Serbsky Russian Federation Ministry of Health, Head Of Department Of Preventive Care In Narcology, Moscow, Russian Federation

**Keywords:** alcohol dependence, self-regulation processes, patients

## Abstract

**Introduction:**

The first stage of the psychological intervention is related to diagnostics

**Objectives:**

Purpose of the study was to explore features of mental self-regulation processes in patients with diagnosis “Mental and behavioral disorders due to use of alcohol”.

**Methods:**

The study involved 39 male patients with alcohol dependence, the average age of 43.6 ± 6 years. The experimental group (20 patients) was taking part in in-patient rehabilitation program, the duration of rehabilitation ranged 4-6 months. The control group included 19 patients of the in-patient addiction treatment department, with average duration of treatment 21 days. To assess self-regulation processes, questionnaires “Style of behavior self-regulation” (Morosanova V.) and questionnaire of volitional self-control (Zverkov A., Eydman E.) were used. To compare differences between two independent groups Mann-Whitney U-test was used

**Results:**

There was a significant difference for the subscale “Persistence and perseverance” in “Volitional self-control” test (p≤0.05) for the control and experimental groups. Patients, involved in clinical rehabilitation program, have higher ranks comparing to patients got clinical treatment (22.2 and 17.7). The comparison of the results of the questionnaire “Style of behavior self-regulation” showed that there is a significant difference for subscales “Modeling of significant conditions” and “Independence” (p≤0.05); participants from the experimental group had higher mean rank in both cases.

**Conclusions:**

Patients who took part in the long-term in-patient rehabilitation program had more stable motivation to achieve their goals, better self-regulation and activity planning skills, higher independence and self-confidence, they were less dependent on opinion of others. The identified features can be used in psychological programs aimed at improving planning skills, reducing behavioral rigidity, stabilizing self-esteem and improving adaptive capacity.

**Disclosure:**

No significant relationships.

